# Exploring Covalent Docking Mechanisms of Boron-Based Inhibitors to Class A, C and D β-Lactamases Using Time-dependent Hybrid QM/MM Simulations

**DOI:** 10.3389/fmolb.2021.633181

**Published:** 2021-08-09

**Authors:** Łukasz Charzewski, Krystiana A. Krzyśko, Bogdan Lesyng

**Affiliations:** Department of Biophysics, Faculty of Physics, University of Warsaw, Warsaw, Poland

**Keywords:** NAMD, covalent docking, boron-based inhibitors, 3-nitrophenyl boronic acid, bicyclic boronate, antibiotic resistance, β-lactamases, time-dependent QM/MM

## Abstract

Recently, molecular covalent docking has been extensively developed to design new classes of inhibitors that form chemical bonds with their biological targets. This strategy for the design of such inhibitors, in particular boron-based inhibitors, holds great promise for the vast family of β-lactamases produced, *inter alia*, by Gram-negative antibiotic-resistant bacteria. However, the description of covalent docking processes requires a quantum-mechanical approach, and so far, only a few studies of this type have been presented. This study accurately describes the covalent docking process between two model inhibitors - representing two large families of inhibitors based on boronic-acid and bicyclic boronate scaffolds, and three β-lactamases which belong to the A, C, and D classes. Molecular fragments containing boron can be converted from a neutral, trigonal, planar state with sp^2^ hybridization to the anionic, tetrahedral sp^3^ state in a process sometimes referred to as *morphing*. This study applies multi-scale modeling methods, in particular, the hybrid QM/MM approach which has predictive power reaching well beyond conventional molecular modeling. Time-dependent QM/MM simulations indicated several structural changes and geometric preferences, ultimately leading to covalent docking processes. With current computing technologies, this approach is not computationally expensive, can be used in standard molecular modeling and molecular design works, and can effectively support experimental research which should allow for a detailed understanding of complex processes important to molecular medicine. In particular, it can support the rational design of covalent boron-based inhibitors for β-lactamases as well as for many other enzyme systems of clinical relevance, including SARS-CoV-2 proteins.

## Introduction

Gram-negative bacteria can cause serious infections in immunocompromised patients, including urinary tract infections, pneumonia, hepatitis, sepsis, soft tissue infections, and peritonitis. It is well-known fact that these bacteria are resistant to such antibiotics as *Penicillin, Cephalosporins, Monobactams,* or *Carbapenems*, because of the ability to breakdown the β-lactam antibiotics by β-lactamases. The reaction mechanism of class A β-lactamases consists of acylation of an active site serine by the antibiotic molecule, followed by deacylation and release of the cleaved compound. Acylation is related to the formation of the tetrahedral intermediate. This reaction mechanism was described in detail using QM/MM and high-level *ab initio* calculations to model the rate-determining step for the formation of a tetrahedral intermediate in acylation (Hermann et al., 2009).

In recent years, significant progress has been made in the design and synthesis of a new class of antibiotics resistant to the β-lactamase mechanisms. In particular, boron-based inhibitors have proven to be effective inhibitors of β-lactamases due to chemical blockade of the tetrahedral transition state forced by these enzymes, and typically due to low toxicity. Boronic acid derivatives, as well as cyclic boronates, have been lately tested in their ability for inhibiting β-lactamases produced, among others, by Gram-negative pathogens *Escherichia coli* and *Klebsiella pneumoniae.* An overview of boron-based small molecules in disease detection and treatment is presented (Theraja, et al., 2017). The combination of theoretical and crystallographic approaches has provided important insight into the molecular stabilization of boron-based compounds in various target proteins, such as proteasomes, tyrosine kinases, histone deacetylases, GPCRs, glutamate racemases, amino acid transporters, autotaxin, and in particular β-lactamases (Calvopiña, et al., 2017; Bello, 2018; Cahill et al., 2019; Song et al., 2021). Based on crystallographic and NMR data, mechanism of proton transfer in class A β-lactamase catalysis and inhibition by *Avibactam* were described (Pemberton et al., 2020).

A number of boron-based chemicals are known as therapeutic agents or drugs, such as *Ixazomib, Tavaborole*, *Crisaborole, Vaborbactam* or *Taniborbactam* (VNRX-5133) Ban et al. (2015), Thareja et al. (2017), Krajnc et al. (2019a), Krajnc et al. (2019b), Lang et al. (2020), Lence and Gonzales-Bello (2021), for specifications see e.g. Drug Central 2021, https://drugcentral.org/. Two authors of this study (Ł.Ch. and K.A.K) participated in integrated studies involving the modeling, synthesis, and biological activity analysis of a large series of boron-based inhibitors of KPC/AmpC β-lactamases (Durka et al., 2019). One should also mention recent work on the evaluation of *Bortezomib* and other boron-containing compounds, as inhibitors of SARS-CoV-2 main protease, e.g. Vega-Valdez et al., 2020). A very comprehensive overview of the mechanisms of β-lactamase inhibition by boron-based inhibitors, including cyclic boronates, and their applications in biomedicine is reported (Brem et al., 2016; Cahill et al., 2017; Cahill et al., 2019; Tooke et al., 2019; Tooke et al., 2020; Song et al., 2021).

**FIGURE 1:**
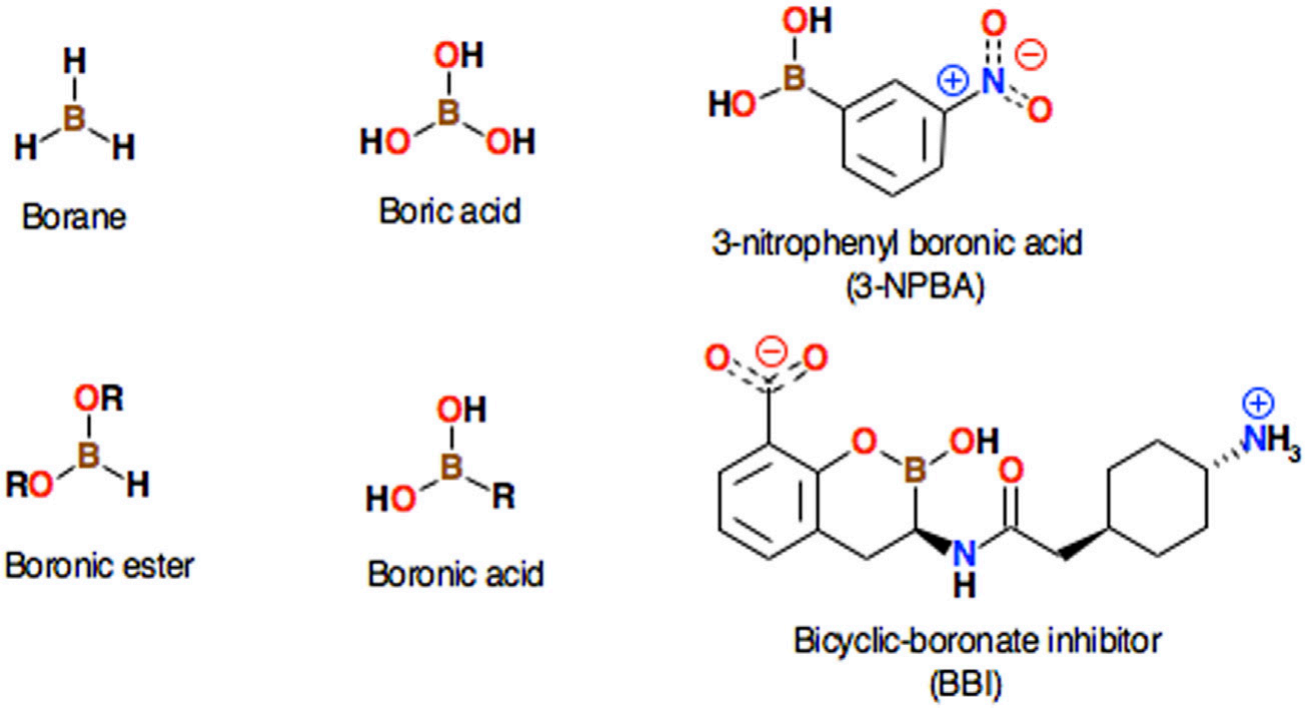
Several well-known simple borane compounds are shown along with their systematic names. The structures of 3-nitrophenylboronic acid (3-NPBA) and the bicyclic boronate inhibitor ((3R)-3-[[2-[4-(amino)cyclohexyl]acetyl]amino]-2-hydroxy-3,4-dihydro-1,2-benzoxaborinine-8-carboxylic acid, BBI), which are the subject of this study, are also presented.

**FIGURE 2:**
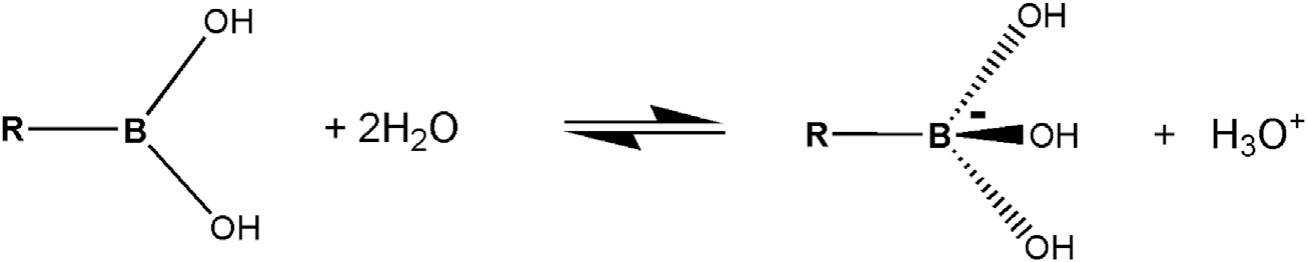
A simplified diagram of the Lewis acid-base model of boronic acid in water.

What is so special about the boron atom? The boron atom constitutes the basis of a huge number of chemical derivatives with very diverse chemical properties; some common borate derivatives including their systematic names are presented (Figure 1). Metalloid boron has many similarities to neighboring carbon and silicon. All three elements form covalent compounds. However, boron has one distinct difference as its outer 2s^2^2p^1^ electronic structure contains one less valence electron than it has valence orbitals. Boron atom is formally trivalent, but due to its empty p-orbital, it can act as a strong acid - the so-called Lewis acid. Its acidic properties will be important for the topic of this work. Lewis acid accepts electrons from electron pair donors, such as H_2_O, HO^−^, NH_3_, or CH_3_^−^. The lone electron pair of the nucleophile interacts with the vacant p-orbital of boron that facilitates the chemical reaction. The interaction of boron with molecular groups having lone electron pairs leads to a tetrahedral transition state, with a valence bond configuration similar to one found in carbon (Figure 2). This way, boronic molecular fragments can easily be converted from the neutral, trigonal planar sp2 hybridization state to the anionic, tetrahedral sp3 state in a process sometimes called *morphing*, which may (or may not) lead to the formation of a chemical bond between boron and the electron donor moiety. It should also be mentioned that in some cases the boron derivatives may also act as Brønsted-Lowry acids. The term *Lewis acid* is more general than *Brønsted-Lowry acid*. In the Brønsted-Lowry model, an acid is characterized as a proton donor and a base as a proton acceptor. The Lewis acid-base model also describes reactions without proton transfer, but where single electron pairs can form new bonds. 3-nitrophenylboronic acid (3-NPBA) and bicyclic boronate derivatives are known covalent inhibitors of β-lactamases (Ke et al., 2012; Brem et al., 2016). 3-NPBA and a bicyclic boronate inhibitor (BBI) being the subjects of this study are shown (Figure 1). 3-NPBA can form a reversible chemical bond with a serine hydroxyl group of the target protein, providing additional covalent-stabilization compared to the initial nonbonded-type interaction, which is the key goal of the discussed activities. The process of formation of such a bond is, however, quite sensitive to the local electric field, depending on the relative orientations of the interacting molecular fragments. In addition, in the case of enzymes, the entire process runs through several transition states that have not been clearly defined so far. Therefore, intentional covalent docking may require a few non-obvious adjustments in order to yield reliable results. Until now, most of the covalent docking work has been supported by heuristic procedures Krajnc et al. (2019a), Krajnc et al. (2019b), and only a few studies of this type of process have been described based on approximate quantum-mechanical methods e.g. (Sgrignani et al., 2015; Krivitskaya and Khrenova, 2021; Lence and Gonzalez-Bello, 2021).

An important problem of boronic acids is their oxidative instability. Phenylboronic acid and its borate esters are oxidized by reactive oxygen species. Quite recently it has been found Graham et al. (2021) that reducing the electron density on the boron increases its oxidative stability. The oxidation of a boronic acid can be slowed down by the presence of a pendant carboxyl group, which is the ligand to the boron atom. The ensuing derivative, oxaborolone, is 10^4^-fold more resistant to oxidation.

Organoboron compounds participate in free radical reactions (Renaud et al., 2007; Kumar et al., 2020). Recent studies describe C-B bond formation mechanisms *via* radical intermediates (Friese and Studer, 2019). Photoactivation processes of boron-species, as well as applications to the creation of C–H, C–C, C–O, B–C, and B–S bond, are discussed in review (Duret et al., 2015). It should be emphasized, however, that as far as the mechanisms of interaction of covalent inhibitors of boron with β-lactamases are concerned, so far there are no experimental or theoretical data that would indicate the radical mechanisms of the formation of chemical bonds of the boron atom with hydroxyl groups of serine residues.

The aim of this work is to accurately describe the covalent docking process between two model inhibitors - representing two large families of inhibitors based on boronic acid and bicyclic boronate scaffolds, and three β lactamases belonging to the A, C, and D enzyme classes, namely KPC-2 β-lactamase (class A), GC1 β-lactamase (class C), and OXA-24 β-lactamase (class D). Class B is not included in this study as it is an ortholog different structurally and mechanistically. As class B catalysis is based on Zn^2+^ ions instead of serine, these metallo-β-lactamases would require a separate study. For a more detailed β-lactamases classification description and properties see e.g. (Bush, 2018; Keshri et al., 2018; Philippon et al., 2016). As mentioned, the first inhibitor is 3-NPBA, and the second one is the inhibitor based on the bicyclic boronate scaffold, named in this study as BBI, (Figure 1). Systematic name of this compound is (3R)-3-[[2-[4-(amino)cyclohexyl]acetyl]amino]-2-hydroxy-3,4-dihydro-1,2-benzoxaborinine-8-carboxylic acid, and it is a close analog of the known *Taniborbactam* antibiotic (Krajnc et al., 2019a; Krajnc et al., 2019b; Liu et al., 2020).

Although the crystallographic structures (6TD1, 6YEN and 6RTN) of *Taniborbactam* complexes with β-lactamases are available Krajnc et al. (2019a), Krajnc et al. (2019b), Lang et al. (2020), Tooke et al. (2020), we decided to use BBI for which such crystallographic data are not available. Among other things, we wanted to prove that the multi-scale modeling and simulation procedures used in this study are so effective that they can be applied to other boron inhibitors of biomedical importance for which direct data from crystallographic or low-temperature electron microscopy of their complexes with protein targets are not available.

## Materials and Methods

In order to accurately simulate the covalent binding process, quantum mechanical and classical molecular mechanical approaches (QM/MM) have to be applied. In particular, a time-dependent molecular dynamics model with a quantum description of structural changes inside the active site should be used, applying quantum-classical molecular dynamics simulations. A number of multiscale molecular modeling and bioinformatics methods are applied in this study, which is briefly discussed below. An overview of multi-scale molecular modeling methods is presented among others in the following reviews Lesyng and McCammon (1993), Lesyng and Rudnicki (2003), Gruziel et al. (2007), and a number of quantum-classical molecular dynamics models along with their numerical implementations are discussed in Bała et al. (1996), Lesyng (1999), Bała et al. (2000), Grochowski and Lesyng (2003). Selected methodologies described above have been used in this study.

Thus, in this study, motions of all atomic nuclei are governed by Newtonian dynamics, and electronic degrees of freedom are described by the evolution of the multi-electronic wave function in an adiabatic way. By adiabatic quantum dynamics, we mean an approximate theoretical model where the wave function of the quantum domain is always in the lowest electronic eigenstate, dependent exclusively on instantaneous positions of the atomic nuclei (molecular geometry based on the Born-Oppenheimer approximation). This approach will hereinafter be referred to as adiabatic quantum-classical molecular dynamics. When it comes to selecting the quantum method, its practical applications in drug design should rather be limited to fast semi-empirical or DFT methods. Within this theoretical framework, taking also into account the accuracy of the QM/MM methods and their computational MD performance, three semiempirical quantum mechanical models PM6, PM7 Stewart (2013), Stewart (2016), and DFTB3 Gaus et al. (2011) deserve attention. PM6 and PM7 have been developed on the basis of the NDDO (Neglect of Diatomic Differential Overlap) approximation. DFTB stands for Density Functional Tight-Binding method. Regarding PM6 and DFTB3 methods, *aposteriori* (D3H4) corrections were designed to improve the hydrogen bond and dispersion interaction energies, Řezáč and Hobza (2012), and Řezáč (2017). The PM6 + D3H4, PM7, and DFTB3 + D3H4 models, as well as a few others, have been tested, including in the analysis non-bonded interactions of inhibitors with enzymes (Križ and Řezáč, 2020).

In this study time-dependent QM/MM simulations were carried out using a high-performance NAMD molecular modeling environment with an implemented quantum potential energy generator MOPAC (Melo et al., 2018; Phillips et al., 2020). In more detail, the NAMD classical environment, VMD visualization environment, and the quantum mechanical package MOPAC Stewart (2016), were integrated into a comprehensive, customizable, and easy-to-use suite. Regarding technical aspects of the hybrid QM/MM model implemented in the NAMD environment, such as definitions of QM regions, mechanical and electrostatic embedding, link atoms, point charge alternations, link atom charge, and charge groups, see http://www.ks.uiuc.edu/Research/qmmm/.

When deciding to use the NAMD environment, the user has a choice of two semi-empirical quantum mechanical models: PM7, or PM6 with a mentioned above *a posteriori* D3H4. While PM6 + D3H4 is slightly better at predicting free energy differences of non-bonded inhibitors interacting with enzymes (Križ and Řezáč, 2020) PM7 has an advantage in predicting structures and heat of formation of chemical bonds. It was parameterized using experimental and advanced *ab initio* reference data, augmented by a new type of reference. In particular, molecular structures containing chemical bonds with boron are slightly better represented in the PM7 model, http://openmopac.net/PM7_and_PM6-D3H4_accuracy/Survey_of_Solids.html/. It has also been validated in the combined docking with classical the MMF94 force field (Sulimov et al., 2017). It should also be noted that time-dependent QM/MM simulations with PM7 Hamiltonian were successfully applied in the theoretical studies of boron-doped derivatives of graphene (Chaban and Prezhdo, 2016).

Therefore, in this work, a hybrid computational model was applied, consisting of the high-performance NAMD computational modeling environment with the classical CHARMM36 force field Vanommeslaeghe et al. (2010), and the MOPAC/PM7 quantum module–serving as the generator of the Born-Oppenheimer potential energy surface.

Structures deposited in Protein Data Bank Berman et al. (2000) of the following β-lactamases: KPC-2 β-lactamase (class A, PDB code 3RXX; Ke et al. (2012)), GC1 β-lactamase (class C, PDB code 1Q2Q; Venkatesan et al. (2004)), and OXA-24 (class D, PDB code 4F94; June et al. (2013)) were studied. With regard to OXA-24 β-lactamase, two forms were considered - wild type, containing carboxylated Lys 84 and its mutant - Lys 84 to Asp 84, denoted hereafter as K84D. The reason for considering the K84D mutant was that we were not able to observe covalent binding in the wild-type. Such a mutation has already been described Schneider et al. (2011), and as indicated the mutant was capable to bind and hydrolyze carbapenems, as well as to covalently bind inhibitors. Considering these biochemical properties and similar electrostatic potential the K84D mutant might effectively mimic the behavior of wild-type.

**FIGURE 3:**
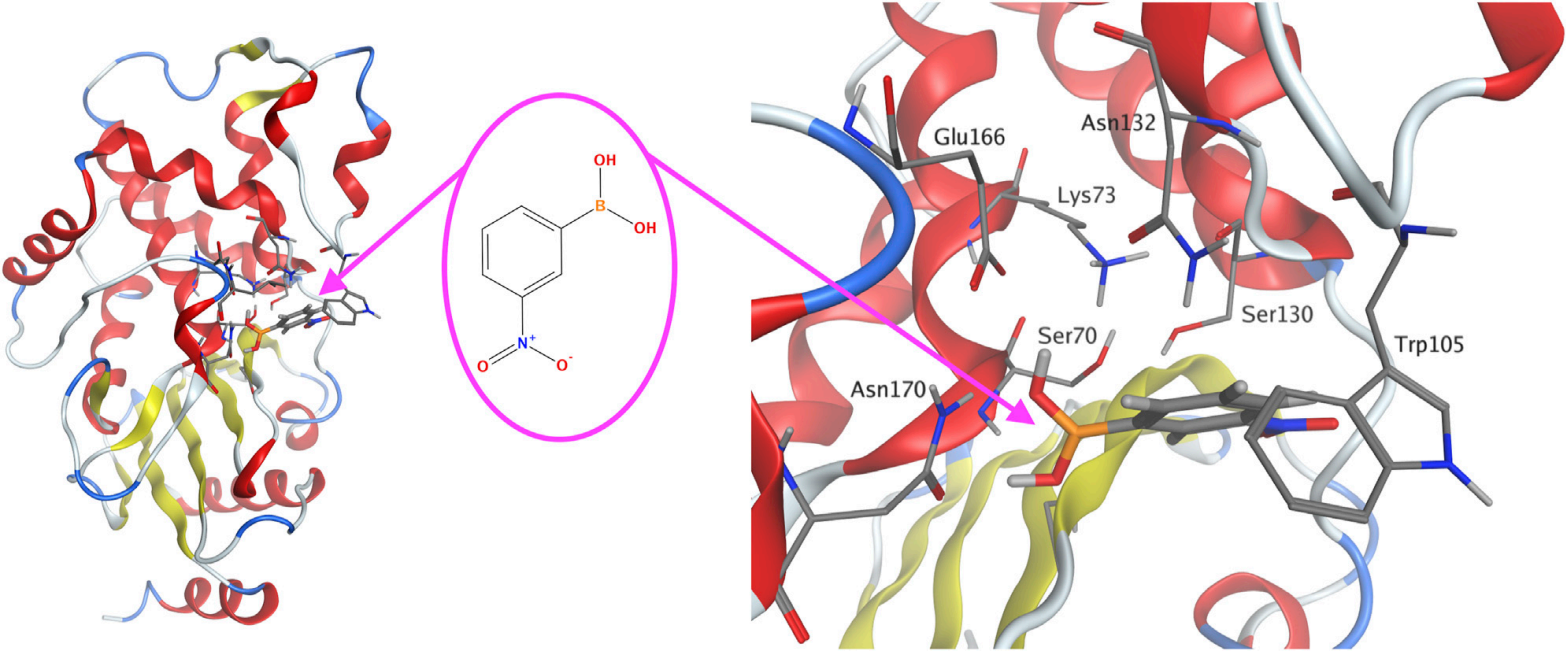
KPC-2 β-lactamase with the docked 3-NPBA substrate molecule **(left)**. The chemical formula of 3-NPBA **(middle)**. The optimized molecular structure with an atomic resolution of KPC-2 β-lactamase focused on the active site with 3-NPBA docked **(right)**. The distance between Ser 70 hydroxyl oxygen and the boron atom of 3-NPBA was 2.57 Ă. The β-lactamase α-helices are shown in red, β-strands in yellow and turns in blue.

The covalent bond between 3-NPBA and Ser 70 was removed from KPC-2 β-lactamase structure, and substrate-state protonation was restored using Molecular Modeling Environment 2019 (MOE, 2019). The structure was optimized by the energy minimization, applying the AMBER10 force field Case et al. (2008) utilizing mild string restraints on aromatic ring carbons in order to preserve ring orientation. Figure 3 presents KPC-2 β-lactamase with the docked 3-NPBA substrate molecule. As finding the structural core of considered proteins and optimizing structural alignment was not trivial, DAMA - a novel multiple structure alignment method was applied (*Essentia Proteomica* server, https://dworkowa.imdik.pan.pl/EP/DAMA/, Daniluk and Lesyng, 2011; Daniluk et al., 2021). Using DAMA, the KPC-2, GC1, and OXA-24 structures were compared, analyzed, and aligned with the identification of the biologically significant similarities. 3-NPBA in an orientation corresponding to the geometry of the optimized KPC-2 β-lactamase complex was introduced to GC1 and OXA-24. The BBI inhibitor was docked in a very similar way as 3-NPBA. Its initial conformation was derived from the structure of *Taniborbactam*. The inhibitor was inserted into the KPC-2, GC1, and OXA-24 binding pockets and oriented similarly to the *Taniborbactam* poses in the crystallographic 6TD1, 6YEN, and 6RTN structures Krajnc et al. (2019a), Krajnc et al. (2019b), Lang et al. (2020), Tooke et al. (2020), respectively. Covalent bonds between serine and the boron atom were removed. The resulting systems were optimized by applying the ligand and pocket sidechains minimization in the AMBER10 force field without any restraints. Next, structures of all complexes were carefully virtually titrated at pH = 7. For titration purposes, we used the generalized Born model Labute (2008), Labute (2009) implemented in Protonate 3D of the MOE modeling environment. The method uses a modified version of the Generalized Born/Volume Integral (GB/VI) formalism for implicit solvent electrostatics. Based on our previous research and experience with generalized Born models Wojciechowski and Lesyng (2004) we judge the above choice as sufficiently precise. The applied procedure resulted in assigning protons to all dissociable functional groups. Protonate 3D includes side-chain rotamer, tautomer, and ionization states of all chemical groups during the course of the free energy minimization of a system. It is very important modeling element in this project. In particular, effective electrostatic interactions determine the zwitterionic structure of the boron-based inhibitors, as well as the protonation states of selected water molecules located in the enzyme active sites. The refined structures were used for the initial conditions to start time-dependent QM/MM simulations using NAMD. Structures were solvated, Na+ and Cl-ions were introduced to neutralize the total charge of the systems and provide ionic strength to 0.05 M. Periodic boundary conditions were applied. The quantum domain consisted of the 3-NPBA (or BBI) inhibitor and all protein residues and water molecules containing at least one atom at a distance smaller than 5 Å from 3-NPBA or 7 Å from BBI. BBI is larger and more elongated than 3-NPBA, therefore its quantum domain has been assumed to be larger. As mentioned, the PM7 Hamiltonian was applied. Prior to productive time-dependent QM/MM simulations, the molecular systems were once more optimized, next thermalized, and equilibrated for 20 ps The productive simulations typically consisted of 20 independent 50 ps runs with a 0.5 fs time-step. The MD trajectories were analyzed using the VMD graphical interface (Humphrey et al., 1996).

The energy barriers for both ligands and all three classes of complexes were estimated applying the steered MD technique (SMD), which has been executed applying the NAMD Adaptive Biasing Forces (ABF) module. For the QM domain, a minimal subset of residues required to construct the reaction pathway has been selected (i.e., only the residues listed in Table 1 and Table 2). The distance bias has been applied to the inhibitor’s boron atom and the target serine hydroxyl oxygen atom (Ser 70, Ser 64, Ser 81 for the A, C, and D classes, respectively). A force constant of 5,000 kcal/mol/Å^2^ has been applied during 5 ps simulations to reduce the B–Ser oxygen distance up to 1.4 Å from that one obtained during the docking procedure. All other conditions were kept exactly the same as in the production runs. Since the propagation of reaction in SMD simulation is significantly slowed down, the QM subsystem energy has been smoothened by the moving average applying a 250 fs window.

**TABLE 1:** Approximate time-periods characterizing the covalent docking process between 3NPBA and the members of the A, C, and D β-lactamases classes. 3-NPBA for simplicity is denoted as NPB. T_arrival_ is the time to reach a given state on the reaction path, calculated from the beginning of the process. T_duration_ is the average time to reach the next state, calculated from the previous one. All times, along with their standard deviations in parentheses, are given in ps. The atomic names follow the CHARMM force field nomenclature as well as the IUPAC nomenclature, and show which atom has moved towards which atom in another molecular fragment. The distances describing the changes in geometry are given in Å. Graphical representations of the reaction steps are shown in Figures 4, 6. In class C, stage #A2 was observed only in the second simulation, reversing step #A1 and leading back to the structure as in step #1. The simulations in class C which were stabilized at stage #A1 resulted in blocking step #2.

Step	Description of the transfer process (CHARMM)	Description of the transfer process (IUPAC)	Change of the distance	T_arrival_			
				First simul.	Second simul.	Third simul.	Average T_arrival_	Average T_duration_
Class A – KPC-2								
#1	Lys73:HZ3 → Glu166:OE2	Lys73:N^6^-H → Glu166:O^5^	2.0 → 1.0	0.20	0.55	0.27	0.34 (±0.15)	0.34 (±0.15)
#2	Glu166 (proton rearrangement)	Glu166 (proton rearrangement)	–	9.05	2.95	24.28	12.09 (±8.97)	11.75 (±9.06)
#3	NPB:B → Ser70:OG	NPB:B^1^ → Ser70:O^3^	2.1 → 1.6	9.35	5.75	24.95	13.35 (±8.33)	1.26 (±1.10)
#4	Ser70:HG → Lys73:NZ	Ser70:O^3^-H → Lys73:N^6^	2.0 → 1.0	9.35	6.98	25.20	13.84 (±8.09)	0.49 (±0.53)
#5	NPB:B → Ser70:OG	NPB:B^1^ → Ser70:O^3^	1.6 → 1.5	10.10	7.00	25.40	14.17 (±8.04)	0.32 (±0.31)
Class C – GC1								
#1	NPB:B → Ser64:OG	NPB:B^1^ → Ser64:O^3^	2.1 → 1.6	0.20	0.15	0.22	0.19 (±0.03)	0.19 (±0.03)
#A1	Lys318:HZ3 → Tyr150:OH	Lys318:N^6^-H → Tyr150:O^4^	1.7 → 1.1	0.67	0.20	0.30	0.39 (±0.20)	0.20 (±0.19)
#A2	Tyr150:HZ3 → Lys318:NZ	Tyr150:O^4^-H → Lys318:N^6^	1.5 → 1.1	–	0.95	–	0.95 (±0.00)	0.75 (±0.00)
#2	Ser64:HG → Tyr150:OH	Ser64:O^3^-H → Tyr150:O^4^	2.5 → 1.0	–	1.35	–	1.35 (±0.00)	0.40 (±0.00)
	NPB:B → Ser64:OG	NPB:B^1^ → Ser64:O^3^	1.6 → 1.5					
Class D – OXA-24, mutant K84D								
#1	NPB:B → Ser81:OG	NPB:B^1^ → Ser81:O^3^	2.1 → 1.6	0.25	0.30	0.43	0.33 (±0.07)	0.33 (±0.07)
#2	H2O:H1 → Asp84:OD2	H_2_O:O-H → Asp84:O^4^	2.0 → 1.0	0.38	0.50	0.55	0.48 (±0.07)	0.16 (±0.04)
	Ser81:HG → H_2_O	Ser81:O^3^-H → H_2_O	2.0 → 1.0					
#3	NPB:B → Ser81:OG	NPB:B^1^ → Ser81:O^3^	1.6 → 1.5	0.43	0.50	0.57	0.50 (±0.06)	0.03 (±0.02)

**TABLE 2:** Approximate time-periods characterizing the covalent docking process between the bicyclic boronate inhibitor (BBI) and the members of the A, C, and D β-lactamases classes. T_arrival_ is the time to reach a given state on the reaction path, calculated from the beginning of the process. T_duration_ is the average time to reach the next state, calculated from the previous one. All times, along with their standard deviations in parentheses, are given in ps. The atomic names follow the CHARMM force field nomenclature as well as the IUPAC nomenclature, and show which atom has moved towards which atom in another molecular fragment. The distances describing the changes in geometry are given in Å. Graphical representations of the reaction steps are shown in Figures 7, 8. In class D, two distinct paths involving one or two H_2_O molecules in step #3 were observed, and are denoted as #A3 and #B3.

Step	Description of the transfer process (CHARMM)	Description of the transfer process (IUPAC)	Change of the distance	T_arrival_			
				First simul	Second simul	Third simul.	Average T_arrival_	Average T_duration_
Class A – KPC-2								
#1	BBI:B → Ser70:OG	BBI:B^2^ → Ser70:O^3^	2.8 → 1.7	0.26	0.37	0.57	0.40 (±0.13)	0.40 (±0.13)
#2	Ser70:HG → H_2_O	Ser70:O^3^-H → H_2_O	2.0 → 1.0	0.28	0.42	0.61	0.44 (±0.14)	0.03 (±0.01)
	H_2_O:H_2_ → Glu166:O	H_2_O:O-H → Glu166:O^5^	1.7 →1.0					
#3	BBI:B → Ser70:OG	BBI:B^2^ → Ser70:O^3^	1.7 → 1.5	0.28	0.42	0.62	0.44 (±0.14)	0.003 (±0.004)
Class C – GC1								
#1	BBI:B → Ser64:OG	BBI:B^2^ → Ser64:O^3^	2.8 → 1.7	0.35	0.32	0.38	0.35 (±0.02)	0.35 (±0.02)
#2	Ser64:HG → Tyr150:OH	Ser64:O^3^-H → Tyr150:O^4^	2.5 → 1.0	0.65	0.35	0.47	0.49 (±0.12)	0.14 (±0.12)
	BBI:B → Ser64:OG	BBI:B^2^ → Ser64:O^3^	1.7 → 1.5					
Class D – OXA-24, mutant K84D								
#1	BBI:B → Ser81:OG	BBI:B^2^ → Ser81:O^3^	2.9 → 1.7	0.45	0.37	0.44	0.42 (±0.03)	0.42 (±0.03)
#2	Ser81:HG → H_2_O(1)	Ser81:O^3^-H → H_2_O(1)	1.7 → 1.0	0.47	0.43	0.47	0.46 (±0.02)	0.03 (±0.01)
#A3	H_2_O(1):H1 → Asp84:OD1	H_2_O(1):O-H → Asp84:O^4’^	1.7 → 1.0	0.55	0.55	–	0.55 (±0.00)	0.07 (±0.05)
#B3	H_2_O(1):H1 → H_2_O(2)	H_2_O(1):O-H → H_2_O(2)	1.7 → 1.0	–	–	0.45	0.45 (±0.00)	0.004 (±0.00)
	H_2_O(2):H1 → Asp84:OD2	H_2_O(2):O-H → Asp84:O^4^	1.7 → 1.0					
#4	BBI:B → Ser81:OG	BBI:B^2^ → Ser81:O^3^	1.7 → 1.5	0.55	0.55	0.57	0.56 (±0.01)	0.04 (±0.01)

Regarding the optimal simulation procedures, the so-called computational assays, for enzymatic activities, including also β-lactamases, several valuable publications dealing with formulation simulation standards for QM/MM methods should be mentioned, in particular (Mulholland, 2005; Fonseca et al., 2012; Van der Kamp and Mulholland., 2013; Chudyk, et al., 2014; Lence, et al., 2018; Hirvonen et al., 2019). Special attention should be devoted to an overview of biomolecular simulations and computational assays (Huggins et al., 2019). However, with regard to covalent docking with boron-based inhibitors, it would be very difficult to directly apply a simulation protocol discussed in the mentioned above works. Also, quite recently an electronegativity equalization method at the σπ level (ABEEMσπ) polarizable force field (ABEEMσπ PFF) of boronic acid and β-lactamase has been developed to determine the potential energy functions and parameters (Lu et al., 2020). This should work for the complex of boronic acid with β-lactamases, and may, however, not be working well for the complex of the bicyclic boronate inhibitor with β-lactamases.

In conclusion, in our opinion, the semi-empirical Hamiltonian PM7 used in this work mimics quite well more advanced *ab initio* methods for enzymatic reactions involving boron atoms. Embedding the QM kernel in the NAMD environment is fully functional and effective. As mentioned, a very important element of this type of modeling is careful titration of the initial molecular state, followed by the energy minimization, thermalization, and equilibration tasks.

## Results

The QM/MM simulations for KPC-2, GC1, and K84D mutant of OXA-24 have successfully reproduced the entire enzymatic process leading to the chemical bond formation between the boron atom of the inhibitor and the hydroxyl group of the catalytic Ser 70 residue (Ser 64 and Ser 81 for GC1 and OXA-24, respectively). The reaction didn’t occur in the case of the wild type OXA-24 enzyme. We suspect that the presence of carboxylated lysine might be connected with some electrostatic/protonation changes, which we were not able to identify and include in the simulations.

**FIGURE 4:**
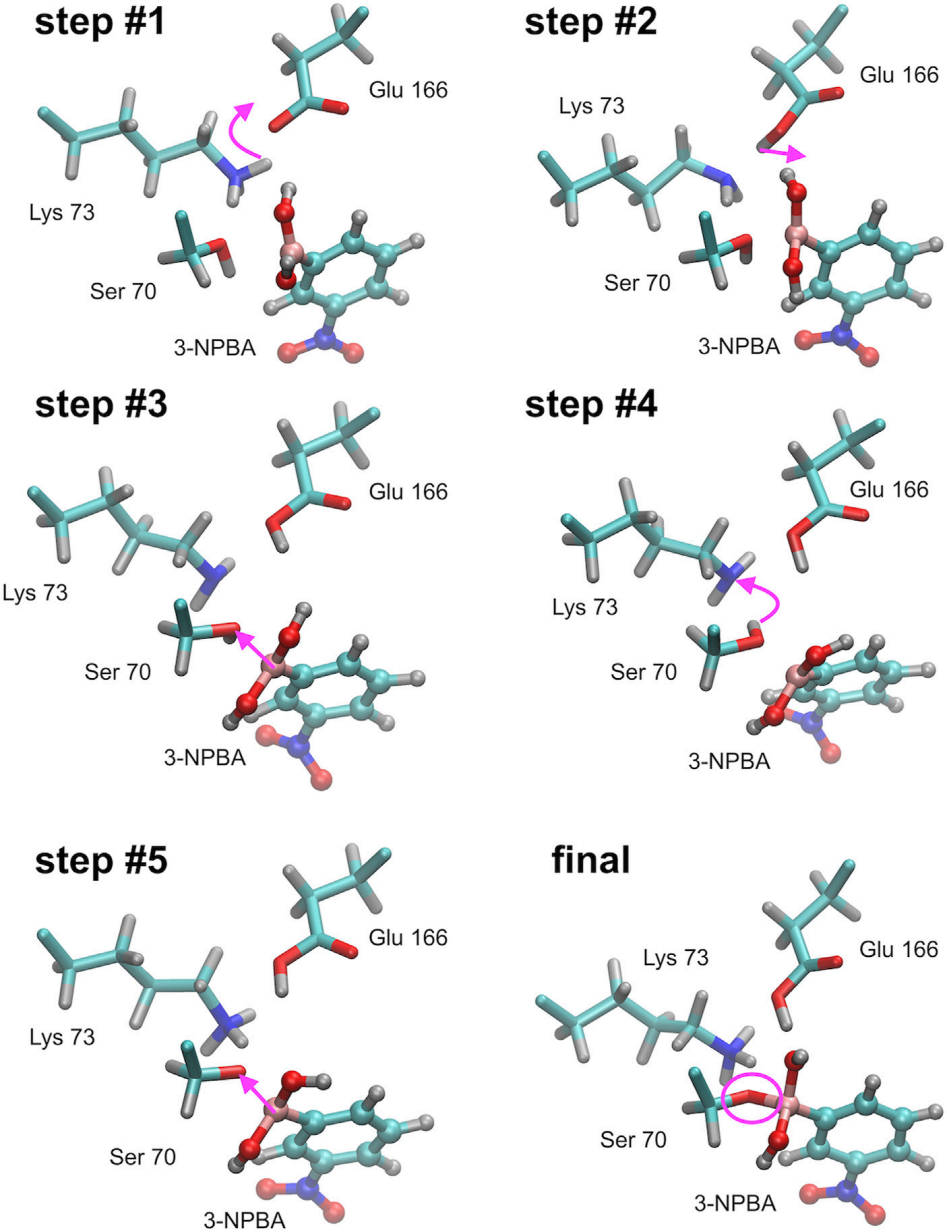
Successive steps of the chemical reaction leading to the bond formation between the Ser 70 hydroxyl group of KPC-2 β-lactamase, and the boron atom of 3-NPBA. Most important molecular and atomic labels are given in Figure 6 (Class A). Curved arrows indicate the proton transfer processes, straight arrows indicate distance reduction. Step #1 – proton transfer from Lys 70 to Glu 166 (Ser 70 - 3-NPBA-boron distance, 2.1 Ă). Step #2 – proton location rearrangement leading to hydrogen bond formation with 3-NPBA-boron (Ser 70 - 3-NPBA-boron distance, 2.1 Ă). Step #3 – the boron atom moves towards the Ser 70 hydroxyl group (Ser 70 - 3-NPBA-boron distance, 1.6 Ă). Step #4 – proton transfer between Ser 70 and Lys 73 (Ser 70 - 3-NPBA-boron distance, 1.6 Å). Step #5 – a movement of the boron atom towards the Ser 70 hydroxyl group (Ser 70 - 3-NPBA-boron distance, 1.5 Å). Final structure–the chemical bond is formed between the hydroxyl oxygen of Ser 70 and the boron atom of 3-NPBA (Ser 70 - 3-NPBA-boron distance, 1.50 Å). Step #4 and step #5 can be described as representatives of the leading transition state on the reaction path.

The simulation results will be more precisely described for the complex of 3-NPBA with KPC-2. The movie of the whole process for this enzyme is available (Supplementary Video S1). During all independent QM/MM simulations the reaction scenario appeared to be the same, indicating intermediate stages and proton transfer processes in the active site. The only differences refer to the time-periods of successive processes. The activation process consists of three steps: first Lys 73 is deprotonated by Glu 166, then one observes rearrangement of the hydrogen bond system in the active site resulting in a hydrogen bond of Glu 166 with one of the hydroxyls of 3-NPBA, and then the key Ser 70 is deprotonated by Lys 73. This immediately allows the formation of the chemical bond between deprotonated hydroxyl of Ser 70 and the boron atom of 3-NPBA. The described above processes are visualized (Figure 4). Note that the hybridization of the boron atom has changed from sp2 (initial state) to sp3 (final state). Simulation correctly predicts changes of the distances between functional groups in the active site, resulting in the final structure very similar to the crystallographic 3RXX structure.

**FIGURE 5:**
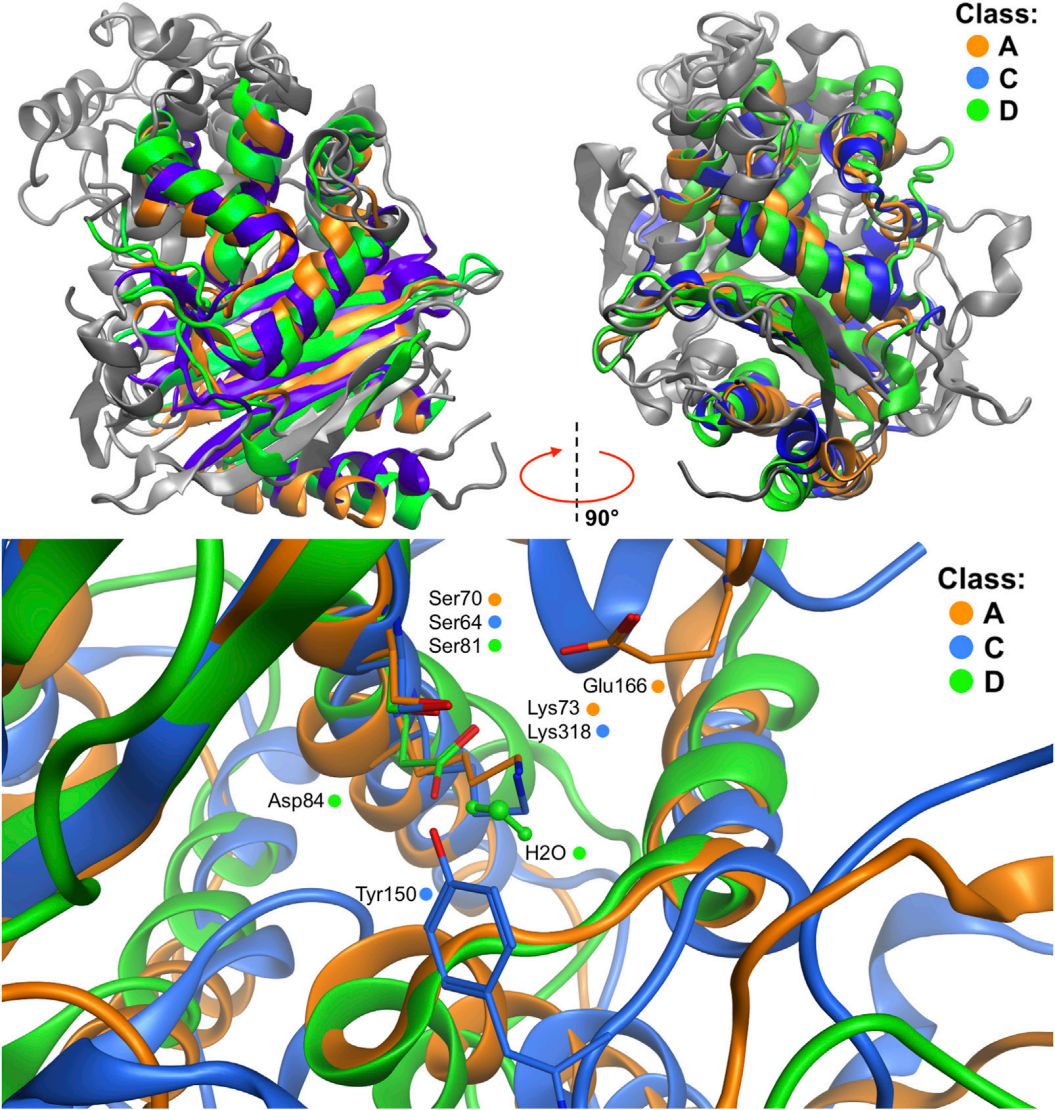
Structural multiple-alignment of the β-lactamases members for the A (KPC-2 β-lactamase), C (CG1 β-lactamase), and D (OXA-24 β-lactamase) enzyme classes. Global alignment with two orientations **(top)**, and presentation of the active sites **(bottom)** resulting from the global alignment. Water residue in class D active site is depicted with balls and sticks representation. Residues crucial in the QM/MM simulations are presented as sticks.

**FIGURE 6:**
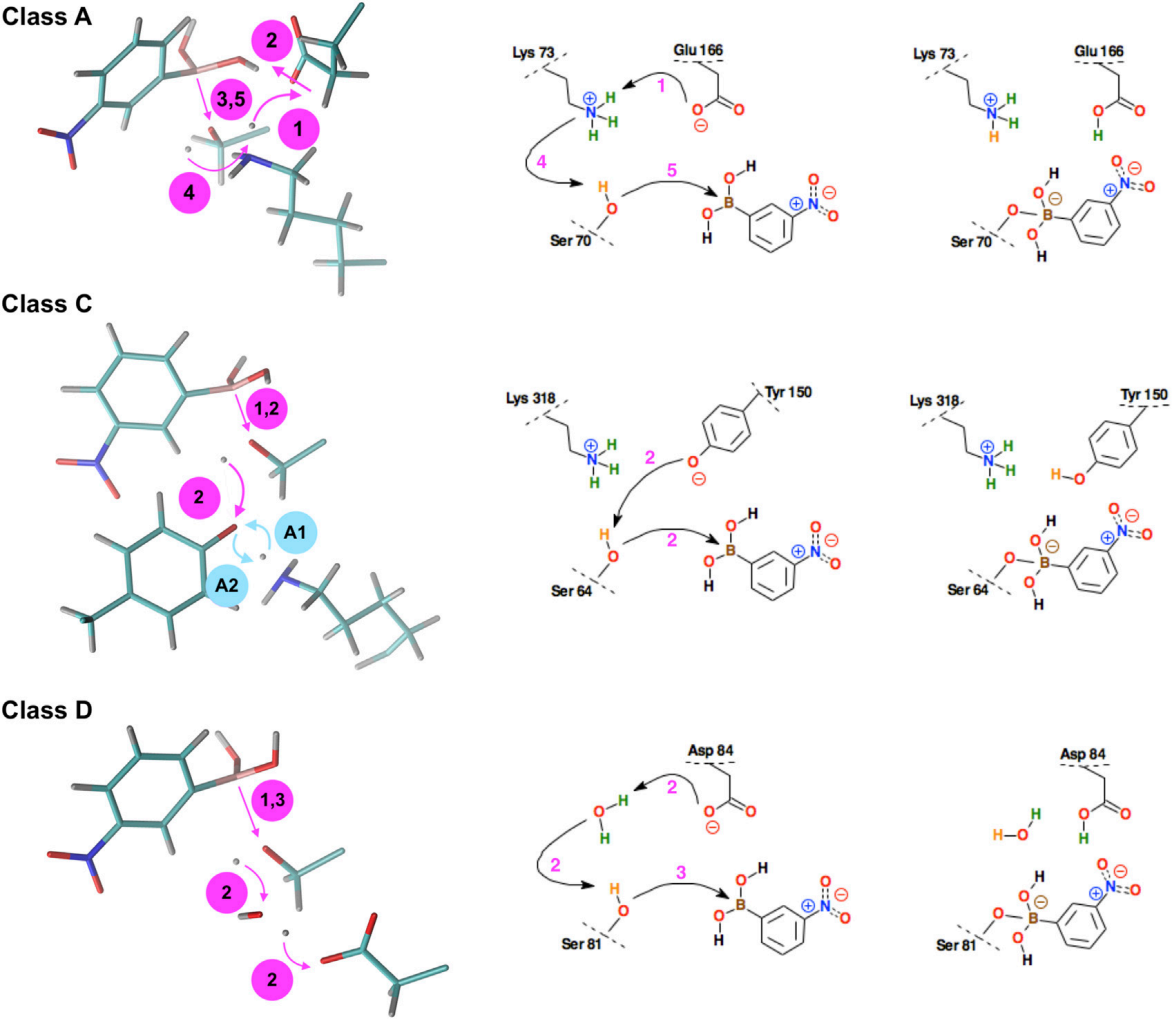
Schematic representation of the successive steps in the formation of the chemical bond between the boron atom of the 3-NPBA inhibitor and the hydroxyl groups of serine β-lactamases. The active site residues belong to KPC-2 β-lactamase (class A), GC1 β-lactamase (class C), and the K84D mutant of OXA-24 β-lactamase (class D). On the left - curved arrows indicate the proton transfer processes, straight arrows indicate distance reduction. In class C, steps #A1 and #A2 (denoted by blue annotations) were observed, however, they are mutually reversible, leading finally to the structure as in step #1. On the right, conventional simplified diagrams showing reaction steps - the arrows symbolically indicate the electron transfer processes. Description of the time-periods related to the presented steps is given in Table 1.

As for the QM/MM simulations of the covalent docking process to representatives of classes C and D β-lactamases, they were preceded by a detailed comparison of the structures of GC1 and OXA-24 with the reference enzyme KPC-2. Global structural multiple-alignment indicated all essential similarities (Figure 5). 10 long, 100 ps, QM/MM simulations of wild-type OXA-24 were carried out in order to check whether there were any unforeseen intuitive processes leading to covalent docking. The simulations, however, did not bring the system out of its initial state. Therefore, similar yet free from post-translational modifications K84D mutant was applied. In this case, covalent docking has proved successful. Scenarios for the covalent docking processes for the members of A, C, and D classes were identified and compared (Figure 6). A detailed description of the covalent docking reaction steps between 3-NPBA and the above-mentioned three β-lactamases, as well as their approximate time-periods, are described (Table 1 **)**. In the case of class C and D β-lactamases, one sees slightly different scenarios for the formation of a chemical bond between the boron atom of 3-NPBA and the oxygen of the Ser 64 (GC1) or Ser 81 (OXA-24) hydroxyl group, compared to class A. Specifically for GC1 β-lactamase, although the productive reaction path is shorter than for KPC-2, there is a possible dead-end involving Lys 318 (Figure 6, blue annotation), which does not lead to the final state, and only withdrawal from it may lead to the effective formation of the above-mentioned bond. In turn, for the K84D mutant of OXA-24 β-lactamase, an important role in the enzymatic process is played by the water molecule from the nearby environment of this enzyme. After deprotonation of the water molecule, the formed hydroxyl anion acts as a very strong base, ultimately leading to the proton dissociation from the hydroxyl group of Ser 81, and effective interaction of the deprotonated hydroxyl group with the boron atom, resulting in the formation of a stable chemical bond -B-O-, as in the previous cases. The most important fragments of the covalent 3-NPBA docking processes to GC1 and OXA-24 are visualized in Supplementary Video S2and inSupplementary Video S3, respectively.

Analyzing the reaction time-periods of the chemical bond formation in 3-NPBA systems, it can be seen that the bottleneck - which in the case of KPC-2 is stage #2, consists of the rearrangement of hydrogen bonds in the active site (11.75 ps). It should be emphasized, however, that there is a significant deviation of times assigned to this process (2.40–24.01 ps), so the average value for the three analyzed trajectories should be treated as a very approximate one. The remaining steps are in all cases very fast and are in the range of 0.20–0.75 ps It should be noted, however, that in the case of GC1 only one third of the trajectories turned out to be productive, at least in terms of the simulation times. If the other two trajectories were significantly lengthened, they might reach the final state, but the average time of the catalytic process would be much longer. Also, one should note that Tyr 150 at the beginning of simulations is in anionic form, according to computational titration results. Similar simulations with neutral Tyr 150 form did not result in the -B-O- bond formation.

**FIGURE 7:**
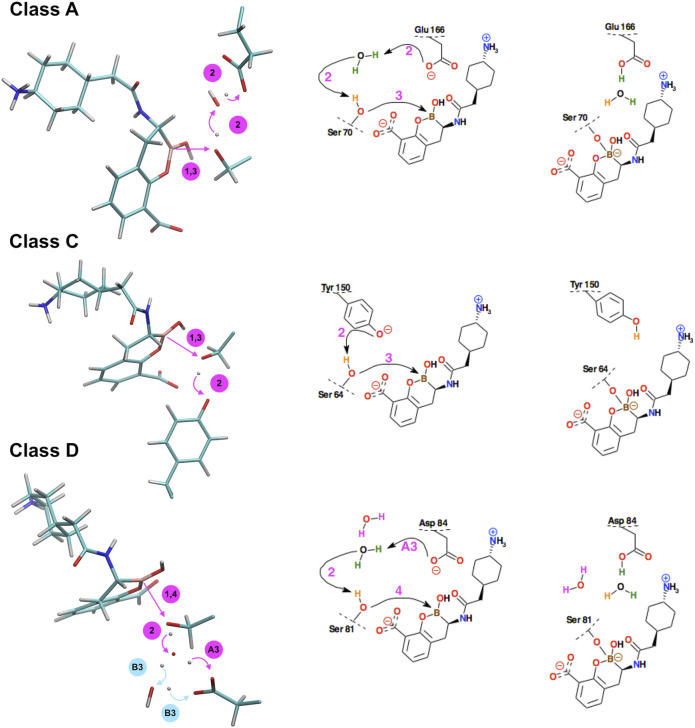
Schematic representation of the successive steps in the formation of the chemical bond between the boron atom of bicyclic boronate inhibitor (BBI) and the hydroxyl groups of serine β-lactamases. The active site residues belong to KPC-2 β-lactamase (class A), GC1 β-lactamase (class C), and the K84D mutant of OXA-24 β-lactamase (class D). Curved arrows indicate the proton transfer processes, straight arrows indicate distance reduction. In class D, steps #A3 and #B3 denote two distinct reaction paths leading to different protonation of Asp84 in the final structure. On the right, conventional simplified diagrams showing reaction steps - the arrows symbolically indicate the electron transfer processes. Description of the time-periods related to the presented steps is given in Table 2.

Regarding covalent docking of the BBI inhibitor, the simulation results are more precisely described for the complex of BBI with KPC-2. Successive steps of the chemical reaction leading to the bond formation between the Ser 70 hydroxyl group of KPC-2 β-lactamase, and the boron atom of BBI are presented (Figure 7). The catalytic site of class A beta-lactamases containing bicyclic boronates contains additional water molecule, which is absent in crystallographic structures of non-cyclic boronates (like 3-NPBA). The presence of this particular water molecule, often called *deacyling water* due to its function in beta-lactam catalysis, suggest that the course of bond formation with BBI might be different. Indeed, the reaction follows a different path, where water mediates the proton transfer form Ser 70 to Glu 166. This pathway is free from the bottleneck identified in 3-NPBA binding. In resulting structure Glu 166 forms hydrogen bond with the water molecule, which in turn forms another hydrogen bond with the ligand’s hydroxyl group (using hydrogen transferred from Ser 70). The movie of the whole covalent docking process for this enzyme is available (Supplementary Video S4).

The covalent docking of BBI to GC1 took exactly the same course as observed in 3-NPBA, however the interference of Lys was not observed in any simulation. BBI docking to OXA-24 follows similar steps as 3-NPBA, which involve one water molecule mediating proton transfer from Ser81 to Asp84. However, an alternative pathway, involving two water molecules, has been observed in this system, which, interestingly, leads to slightly different final state. The proton transferred through both water molecules binds to different oxygen of Asp 84 carboxyl group.

Most important fragments of the covalent BBI docking processes to GC1 are visualized inSupplementary Video S5, and to OXA-24 in Supplementary Video S6as well as Supplementary Video_7 (two reaction paths for OXA-24, identified). A detailed description of the covalent docking reaction steps between the BBI covalent inhibitor and the above-mentioned three β-lactamases, as well as their approximate time-periods, are described (Table 2). Interestingly, the intermediate Ser oxygen–boron distance, in which the system waits for proton transfers to take place, is 0.1 Ă shorter in BBI.

Due to the almost immediate timescale of the reaction steps, SMD simulations were used to shed a light on the energy profile of the reaction pathways. A few energy barriers has been observed. One should stress, however, the following. The dynamics of the reaction site is highly cooperative. In the case under study, the surface of the chemical reaction consists of a series of narrow maxima in the configuration space, that are traversed as a result of the strong coupling of the reaction site with the dynamics of the enzyme as a whole. In such a situation, an arbitrary choice of a one-dimensional reaction path in the SMD process must always lead to an overestimation of the energy barriers. The classical Potential of Mean Force (PMF) in this case is not a well-defined physical property. After all, the system does not have to choose the reaction path that we indicated, because the path is multidimensional, and we significantly disturb the natural dynamics of enzymes. The one-dimensional choice is justified when it concerns, for example, the slow degree of freedom. Although in this case, we chose a fairly natural reaction path, which is the distance between the boron atom and the oxygen of the serine hydroxyl group, it is probably not the slowest degree of freedom, because the boron atom strongly interacts with the oxygen atom. To summarize the applied SMD procedure gives us only an upper estimate of the PMF - the real barriers are smaller. Below are the upper estimates.

In the case of 3-NPBA ligand, a barrier below 11 kcal/mol occurs between 1.5–4.0 ps (B–Ser:O distance: 2.2 → 1.7 Å) for class A, below 7 kcal/mol between 1.6–3.0 ps (2.6 → 1.9 Å) for class C, and below 5 kcal/mol between 1.0–1.8 ps (2.3 → 2.1 Å) for class D.

For BBI a barrier below 9 kcal/mol occurs between 0.2–2.2 ps (B–Ser:O distance: 2.8 → 2.2 Å) for class A, below 12 kcal/mol between 0–2.5 ps (2.8 → 2.1 Å) for class C, and below 12 kcal/mol between 1.0–3.0 ps (2.7 → 2.0 Å) for class D. Representative energy profiles for each system are illustrated in Supplementary Figures S3, S4).

It should be added that in real systems, such as those studied in this paper, most likely quantum tunneling effects play an indispensable role due to cooperative proton transfer processes. This can lower the barriers even further. These effects can be accounted for by applying quantum dynamics, based explicitly on the time-dependent Schroedinger equation. This type of physics was, inter alia, used in a full description of the phospholipase A_2_ reaction mechanism and model proton transfer systems Bala et al. (1996), Lesyng (1999), Bala et al. (2000), Grochowski and Lesyng (2003). This requires, however, much faster than PM6, PM7 or DFTB3 generators of the potential energy surface.

## Discussion

Multiscale molecular modeling methods have been applied in covalent docking studies using two boron-based inhibitors (3-NPBA and BBI, representing two large families of inhibitors, characterized by boronic acid and bicyclic boronate scaffolds), and three serine β-lactamases (KPC-2, GC1 and OXA 24, representing the A, C and D enzyme classes). This resulted in six successful covalent docking events and seven reaction paths (two paths identified for OXA-21). It should be noted that due to the presence of the functional -NO2 group in 3-NBPA, and -COO- in BBI the inhibitors are quite reactive.

In the main thread of this research, the hybrid, quantum-classical molecular mechanics and adiabatic quantum-classical molecular dynamics model were applied. The DAMA and MOE modeling environments were used in the analysis of the biomolecular structures, virtual titration, and their preparation to the simulations of the covalent docking processes. The QM/MM model implemented in the NAMD/VMD environment, with the approximate PM7 Hamiltonian describing the quantum domains of the enzymes, appeared to be a very effective approach to simulating the covalent docking processes. From the computational point of view, and with the current state of development of computing techniques, adiabatic time-dependent QM/MM simulations are fully acceptable for practical applications for molecular design or drug design experiments. 3D visualization with VMD and MOE tools, based on accurate physical simulations of this enzymatic process, provided detailed information on the very specific role of active site amino acid residues, water molecules and boron-containing molecular fragments, in the covalent docking process. Class B, which contains significantly different metallo (Zn^+2^) β-lactamases, has not been studied.

The covalent 3-NPBA docking mechanism to KPC-2 has been described in great detail. Other systems with less accuracy, but the attached diagrams, tables and videos also allow to recreate all the details. In particular, results of the QM/MM simulations of the covalent reactions between the boron atom and hydroxyl of Ser 81 are well documented in a form of seven movies available in “Supplementary Material” (Supplementary Video S1. MOV, 3-NPBA–KPC-2; Supplementary Video S2. MOV, 3-NPBA–GC1; Supplementary Video S3. MOV, 3-NPBA–OXA 24; Supplementary Video S4. MOV, BBI–KPC-2; Supplementary Video S5. MOV, BBI–GC1; Supplementary Video S6. MOV and Supplementary Video S7. MOV present two reaction paths for the BBI–OXA 24 complex).

In the case of class D for the wild-type enzyme, the formation of the required -B-O- bond was not achieved due to the presence of a carboxylated Lys 84 derivative in the active site. However, it was shown that the K84D mutant very easily leads to the formation of the expected bond due participation of a water molecule from the solvent, which because of the proton dissociation acts as a strong catalytic base initiating the productive reaction path. The positioning of carboxylated Lys 84 in the active site suggests that in a wild-type system, the Ser 81 hydrogen transfer may occur directly to Lys carboxyl oxygen. For members of class A and C, deprotonated Lys 73 and Lys 318, act as such bases, respectively.

To the best of our knowledge, this study is the first one that describes in detail, the course of the covalent docking processes resulting from the quantum description of structural changes in the enzyme active sites, and with the participation of the 3-NPBA inhibitor with the boronic acid scaffold (Figures 3–6 and Table 1), as well as the BBI inhibitor with the bicyclic boronate scaffold (Figure 7 and Table 2). It should be noted that in class D, one or two water molecules play a key role in the deprotonation of Ser 84, which is a prerequisite for the final reaction to form a covalent bond between the boron atom and serine oxygen. The role of these water molecules can be related to the concept of “flip-flop” hydrogen bonds, and/or “water wires”, which have been observed in many biomolecular systems, including enzyme systems. For a review of the first experimental observations and quantum-mechanical analysis of the “flip-flop” hydrogen bonds see (Saenger et al., 1985; Jeffrey and Saenger, 1991). Below are some representative publications analyzing the mechanisms of delocalized hydrogen bonds and “water wires” (Cui and Karplus, 2003; Cukierman, 2003; Marx, 2006; Yan et al., 2007; Sakti et al., 2019; Setny, 2020). The analysis of structural conformational changes coupled with proton transfer processes shows that the covalent docking reactions described in this study are highly cooperative processes, and future studies should probably also take into account the quantum-dynamical effects associated with cooperative proton transfer mechanisms.

In order to better understand electron charge rearrangements during the covalent docking processes, the Mulliken atomic charges have been computed as functions of time. These data are presented in Supplementary Figures S3, S4. There is no indication that any larger electron transfer processes assigned to protons take place. Atomic charges are quite typical and to our surprise, changes of the atomic charges assigned to protons are small. In conclusion, radical reactions involved in covalent docking mechanisms of boron-based inhibitors are highly unlikely - at least for the A, C, and D β-lactamases.

**FIGURE 8:**
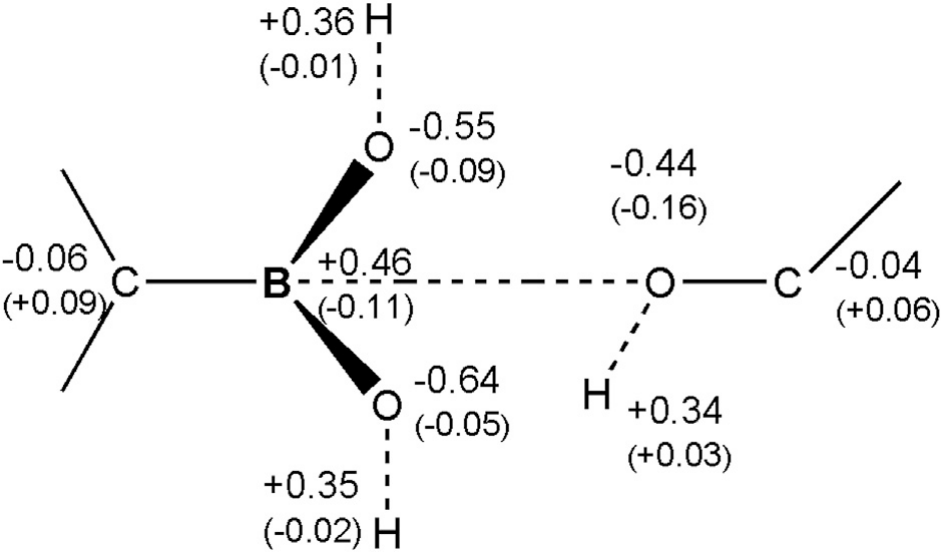
Atomic Mulliken charges on the boron molecular fragment of the 3-NPBA inhibitor, as well as on the hydroxyl group of Ser 70. The charges were computed for the initial state, before the formation of the chemical -B-O- bond. In parentheses are changes of the charges for the final state, after the formation of the chemical bond.

Changes of Mulliken atomic charges, related especially to the change of the hybridization state of atomic orbitals on the boron atom, from the sp^2^ to sp^3^ type and formation of the -C-B-O- bond are also presented (Figure 8). Boron is the electropositive atom in comparison to the oxygen atom. Before the formation of the chemical bond with oxygen in 3-NPBA system, its Mulliken charge was about +0.46 e. After the reaction, this charge drops down to about +0.35 e. In turn, oxygen of the serine hydroxyl group, after creating the chemical bond, increases its negative charge from −0.44 e to −0.60 e, and thus becomes similar to the other two oxygen atoms chemically bonded to the boron atom. In BBI systems similar boron charge drop was observed, from the +0.49 e to +0.32 e after bond formation. Serine oxygens charge however acted differently and changed from −0.68 e to −0.54 e acquiring similar charge to the heterocyclic oxygen. The charge on oxygen in the BBI boron-attached hydroxyl group changed from −0.70 e to −0.48 e.

The energy profiles for the 3-NPBA–KPC-2 and BBI–KPC-2 complexes were assessed by monitoring the energy changes of the QM subsystem consisting of the residues and the inhibitors directly involved in the reaction, as well as by applying SMD simulations. Visual analysis of the SMD trajectories suggests that the observed energy barriers for both 3-NPBA and BBI in each β-lactamase class are amongst others associated with the tension created by bending boron substituents out of the primary trigonal plain when approaching the serine oxygen atom. We think that right after the system breaks this barrier the boron atom undergoes the morphing process, and the sp^3^-associated tetragonal geometry relaxes this tension, resulting in a further energy decrease.

The analysis of structural changes in the enzyme active sites, involving also the 3-NPBA inhibitor, showed during the time-dependent QM/MM simulations several geometric preferences finally leading to the covalent docking processes. Thus, the distance between the boron atom and the oxygen atom of the serine hydroxyl group should be in the order of 3 Å, or slightly less before the chemical bond is formed. This distance can be compared to the sum of the Pauling atomic radii of those atoms, which are 1.65 and 1.42 Å, respectively. The resulting distance, due to long-range non-bonded interactions, and following covalent interactions, rapidly decreases. It should be emphasized that the nonbonded interactions after molecular structure optimization reduce this distance up to 2.6 Å (or to 2.8 Å for bicyclic boron inhibitors). The orientation of the inhibitor’s C-B bond in relation to the HO-C serine bond, defined by the C-B-O angle should be close to 90°. It gives the possibility of the interaction of the “empty” 2p_z_ orbital of boron in its initial sp^2^ hybridization, with the 2p_z_ orbital of oxygen. Deprotonation of the hydroxyl group allows donation of electrons to the boron atom and its transition to the sp^3^ hybridization, thus increasing this angle to the range of 105–120°. After the formation of the chemical bond, the distance between the boron atom and the serine oxygen atom drops down to about 1.5 Å. As already noted, there should be at least one fairly strong basic group in the active site that will be able to sequentially deprotonate the hydroxyl groups bound to the boron atom, as well as the hydroxyl group of serine.

In order to obtain the highly specific effects of boron-based inhibitors, care must be taken that the optimal orientation of the boron atom to a particular serine group is correlated with as many other local interactions as possible. Such interactions can be hydrogen bonds, salt bridges, or localized hydrophobic interactions. The idea is that the chemically active inhibitor should only bind in a highly specific manner to a selected binding site belonging to a particular protein, and therefore that it cannot attack other hydroxyl groups, which could lead to serious side effects.

Presented results allow for a much deeper understanding of the complex covalent docking processes important for molecular medicine, related in particular to the design of novel drugs. They should support the rational design of covalent boron-based inhibitors for β-lactamases, which are the weapon of antibiotic-resistant bacteria, as well as for many other enzyme systems of clinical relevance.

## Data Availability

The original contributions presented in the study are included in the article/Supplementary Material, further inquiries can be directed to the corresponding author.
