# Supplementary material for: Exploring Covalent Docking Mechanisms of Boron-Based Inhibitors to Class A, C and D β-Lactamases Using Time-dependent Hybrid QM/MM Simulations
Source: Front Mol Biosci. 2021 Aug 9;8:633181. doi: 10.3389/fmolb.2021.633181 (PMC8380965; doi:10.3389/fmolb.2021.633181)
Supplement: Supplementary file 1 [file DataSheet2.PDF]

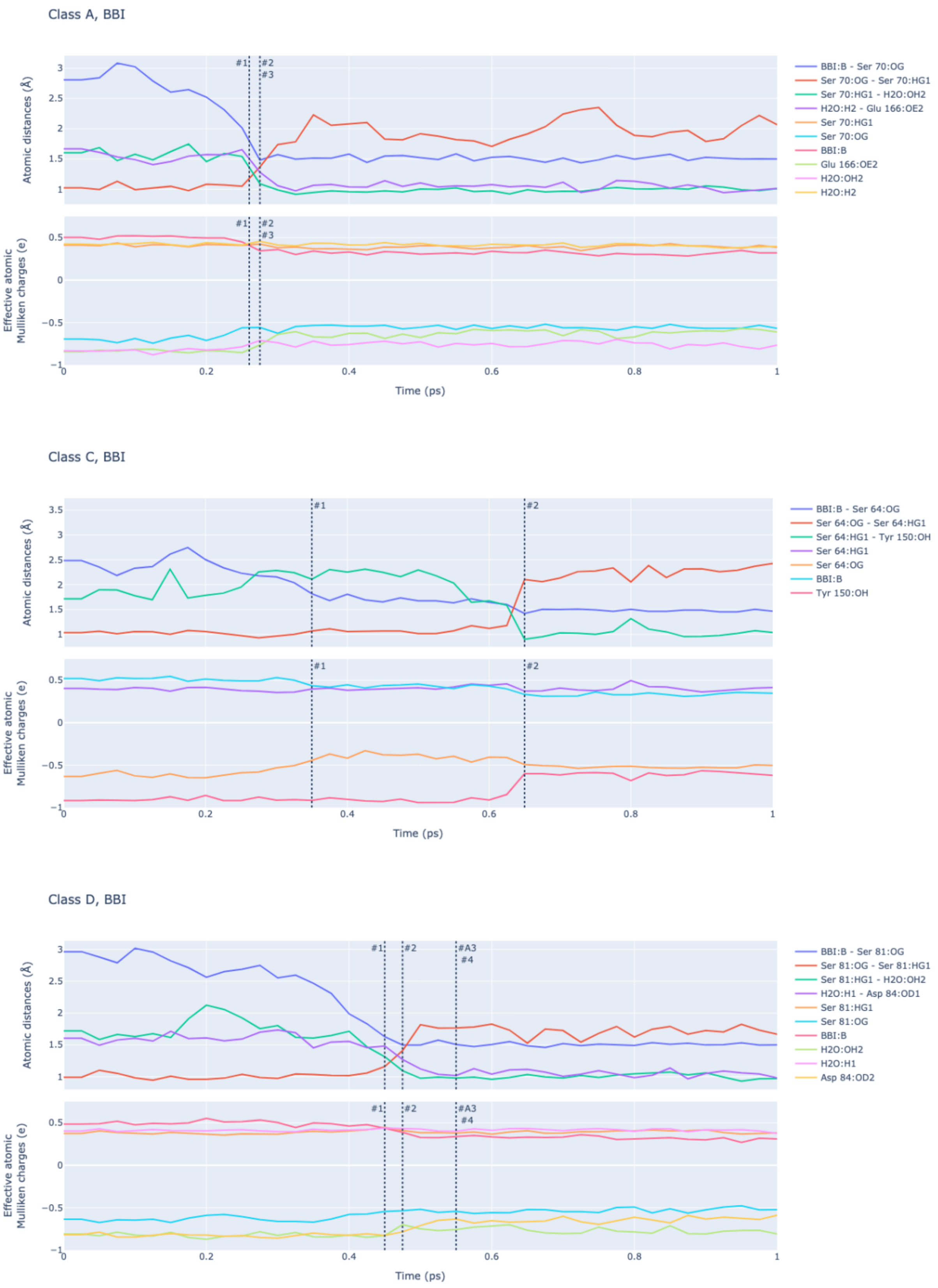

Figure S2. Atomic distances (upper subplots), and effective Mulliken atomic charges (lower subplots) of atoms directly involved in the reaction pathway, presented for BBI docked to β-lactamases which

belong to the A, C, and D classes, respectively. The simulation results in the plots are based on the “first simulation” described in **Table 2**. The reaction steps, marked with vertical dashed lines, correspond to denotations in **Table 2** and in **Figure 7**.
