# Supplementary material for: Exploring Covalent Docking Mechanisms of Boron-Based Inhibitors to Class A, C and D β-Lactamases Using Time-dependent Hybrid QM/MM Simulations
Source: Front Mol Biosci. 2021 Aug 9;8:633181. doi: 10.3389/fmolb.2021.633181 (PMC8380965; doi:10.3389/fmolb.2021.633181)
Supplement: Supplementary file 4 [file DataSheet4.PDF]

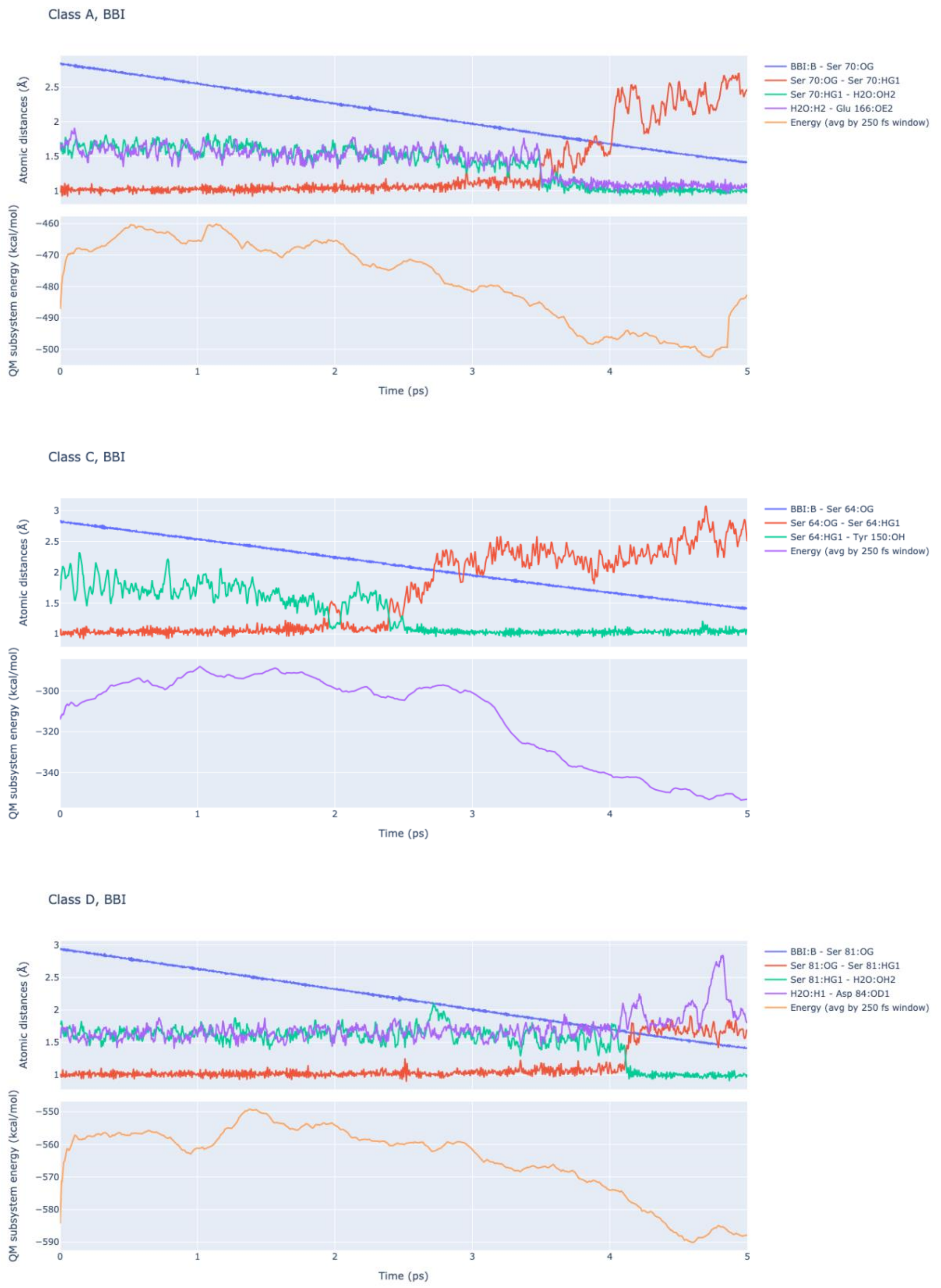

Figure S4. Steered molecular dynamics (SMD) simulations results for the complexes of BBI and β-lactamases which belong to the A, C, and D classes, respectively. The distance between the boron atom of 3-NPBA and the oxygen atom of Ser OG was gradually reduced. Atomic distances (upper subplots)

and the QM subsystem energy of the residues and the inhibitor directly involved in the reaction (lower subplots).
