# Supplementary material for: Exploring Covalent Docking Mechanisms of Boron-Based Inhibitors to Class A, C and D β-Lactamases Using Time-dependent Hybrid QM/MM Simulations
Source: Front Mol Biosci. 2021 Aug 9;8:633181. doi: 10.3389/fmolb.2021.633181 (PMC8380965; doi:10.3389/fmolb.2021.633181)
Supplement: Supplementary file 7 [file DataSheet1.PDF]

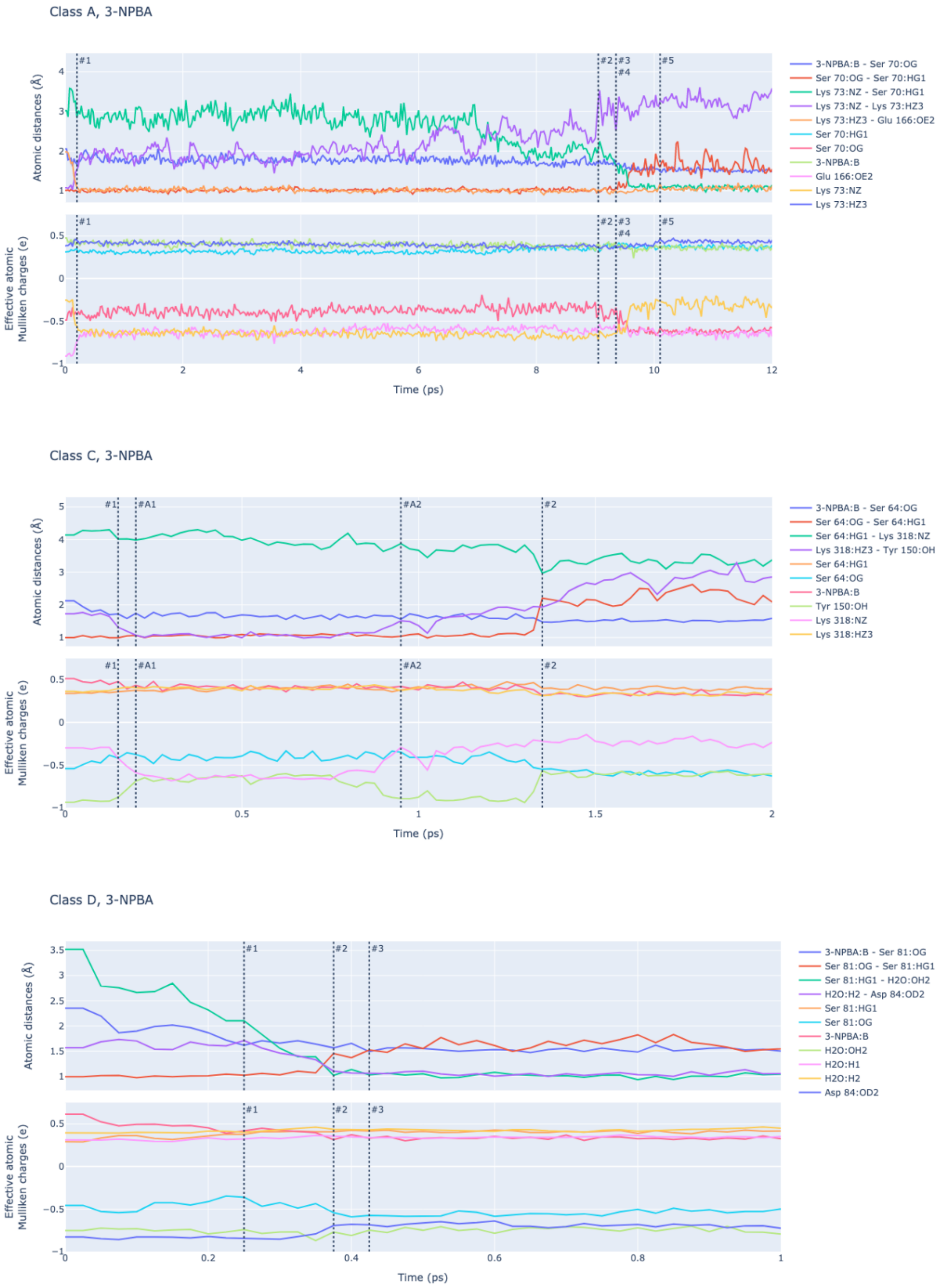

Figure S1. Atomic distances (upper subplots), and effective Mulliken atomic charges (lower subplots) of atoms directly involved in the reaction pathway, presented for 3-NPBA docked to β-lactamases which belong to the A, C, and D classes, respectively. The simulation results in the plots are based on the "first simulation" described in **Table 1**, except the class C which is represented by the "second simulation"

(due to non-productive completion of the first one). The reaction steps, marked with vertical dashed lines, correspond to denotations in **Table 1** and in **Figure 6**.
